# Carbapenemase VCC-1–Producing *Vibrio cholerae* in Coastal Waters of Germany

**DOI:** 10.3201/eid2310.161625

**Published:** 2017-10

**Authors:** Jens A. Hammerl, Claudia Jäckel, Valeria Bortolaia, Keike Schwartz, Nadja Bier, Rene S. Hendriksen, Beatriz Guerra, Eckhard Strauch

**Affiliations:** German Federal Institute for Risk Assessment, Berlin, Germany (J.A. Hammerl, C. Jäckel. K. Schwartz, N. Bier, B. Guerra, E. Strauch);; Technical University of Denmark, Lyngby, Denmark (V. Bortolaia, R.S. Hendriksen)

**Keywords:** carbapenemase VCC-1, carbapenem resistance, antimicrobial resistance, Vibrio cholerae, bacteria, whole-genome sequencing, multilocus sequence typing, sequence type, environment, coastal water, Germany, Europe

## Abstract

During antimicrobial drug resistance testing for *Vibrio* spp. from coastal waters of Germany, we identified 4 nontoxigenic, carbapenem-resistant *V. cholerae* isolates. We used whole-genome sequencing to identify the carbapenemase gene *bla*_VCC-1_. In addition, a molecular survey showed that more *bla*_VCC-1_–harboring isolates are present in coastal waters of Germany.

Mangat et al. recently identified a novel ambler class A carbapenemase (VCC-1) in nontoxigenic *Vibrio cholerae* isolated from imported retail shrimp from India intended for human consumption in Canada (*1*). Occurrence of *bla*_VCC-1_–harboring bacteria in seafood might be caused by uptake of *V. cholerae* in the aquatic environment. Lutz et al. reported worldwide distribution of *V. cholerae* non–O1/O139 strains in coastal waters with low salinity (*2*). Some of these strains were associated with wound infections and with diarrheal diseases after ingestion of contaminated seafood (*3*,*4*).

An antimicrobial resistance survey of potentially pathogenic *Vibrio* spp. recovered from coastal waters of Germany identified 4 carbapenem-resistant *V. cholerae* non–O1/O139 isolates (*5*). These isolates were detected in the Baltic Sea (VN-2997, Eckernförde) and North Sea (VN-2808, Büsum; VN-2825, Speicherkoog; VN-2923, unknown). We used whole-genome sequencing to examine the genetic basis of carbapenem resistance in these strains.

We isolated genomic DNA by using the Easy-DNA Kit (Invitrogen, Carlsbad, CA, USA). This DNA was used for preparation of libraries by using the Nextera-XT-DNA Sample Preparation Kit (Illumina Inc., San Diego, CA, USA) and sequenced by using an MiSeq-benchtop-sequencer, MiSeq-Reagent version 2 (300 cycles), and two 150-bp paired-end reads (Illumina Inc.). We then performed de novo assemblies of reads by using SPAdes version 3.5.0 (http://spades.bioinf.spbau.ru/release3.5.0/manual.html). We deposited sequences in GenBank (Table) and preformed genome annotation by using the NCBI Prokaryotic Genome Annotation Pipeline (https://www.ncbi.nlm.nih.gov/genome/annotation_prok/).

**Table:** Genome characteristics of 4 carbapenemase-producing *Vibrio cholerae* isolates from coastal waters of Germany*

Characteristic	Isolate			
	VN-2808	VN-2825	VN-2923	VN-2997
No. genes	3,934	3,921	4,040	3,933
Coding genes	3,545	3,608	3,650	3,612
No. CDS	3,813	3,803	3,920	3,814
Coding CDS	3,545	3,608	3,650	3,612
No. RNA genes	121	118	120	119
No. rRNAs (5S, 16S, 23S)	10, 12, 7	10, 12, 4	10, 11, 7	10, 12, 4
Complete rRNAs (5S, 23S)	10, 1	10, 1	10, 1	10, 1
Partial rRNAs (16S, 23S)	12, 6	12, 3	11, 6	12, 3
No. tRNAs	88	88	88	89
No. noncoding RNAs	4	4	4	4
No. pseudogenes	268	195	270	202
Ambiguous residues	0	0	0	0
Frameshifted	39	37	40	37
Incomplete	220	144	221	152
Internal stop	20	25	22	23
Multiple problems	11	11	13	10
No. predicted prophages	5	2	5	2
Intact	2	0	1	0
Incomplete	3	1	4	2
Questionable	0	1	0	0
No. plasmids	ND	ND	ND	ND
Antibiotic resistance				
β-lactam†	*bla*_VCC-1_ (100%)	*bla*_VCC-1_ (100%)	*bla*_VCC-1_ (100%)	*bla*_VCC-1_ (100%)
MLST type	ST336	ST336	ST336	ST336
GenBank information				
Bioproject no.	PRJNA331077	PRJNA331078	PRJNA331079	PRJNA331080
Biosample no.	SAMN05437226	SAMN0537225	SAMN0537224	SAMN0537223
Accession no.	MCBB00000000	MCBA00000000	MCAZ00000000	MCAY00000000

*In silico analyses were conducted by using the services of the NCB Prokaryotic Genome Annotation Pipeline (https://www.ncbi.nlm.nih.gov/genome/annotation_prok/), the Center for Genomic Epidemiology (http://.genomicepidemiology.org), and PHAST (http://www.phast.wishartlab.com/).CDS, coding DNA sequence; MLST, multilocus sequencing typing; ND, not determined; ST, sequence type. †Percentage values indicate level of nucleotide similarity against the reference gene (GenBank accession no. KT818596).

We found *bla*_VCC-1_ in all isolates on contigs of 2,135 bp (VN-2825, VN-2997), 2,139 bp (VN-2923), and 2,737 bp (VN-2808). The *bla*_VCC-1_-coding sequences and flanking nucleotide sequences were 100% identical among the strains, as determined by sequence alignments. We also identified the main characteristics of *V. cholerae* genomes (Table; Technical Appendix Figure). Overall, the genomes belong to the same multilocus sequence type (ST), ST336 (*adk* 57, *gyrB* 76, *mdh* 14, *metE* 115, *pntA* 18, *purM* 1, *pyrC* 101) (*6*). 

We performed functional studies of the entire *bla*_VCC-1_–harboring region (pVCC-1C, 2.7 kb) and *bla*_VCC-1_ gene (pVCC-1G, 0.9 kb) of *V. cholerae* VN-2808 (Technical Appendix Figure) by molecular cloning of PCR-amplified regions according to standard procedures (*7*). After transformation of *Escherichia coli* GeneHogs (Invitrogen, Darmstadt, Germany) and susceptibility testing for aztreonam, imipenem, and meropenem as described (*5*), both constructs showed decreased inhibition zone diameters compared with that for *E. coli* GeneHogs vector. We observed slightly reduced drug susceptibility levels or intermediate resistance levels, as observed in *V. cholerae* VN-2808 (Technical Appendix Figure).

On the basis of these results, we conducted *bla*_VCC-1_ screening of the *V. cholerae* collection at the German Federal Institute for Risk Assessment (Berlin, Germany). This collection contains 312 toxigenic and nontoxigenic isolates from human, environmental, and food origin obtained in Europe (n = 218), Africa (n = 20), Asia (n = 18), North America (n = 1), South America (n =1), and of unknown origin (n = 54) during 1941–2015.

We performed PCR by using primers (bla_VCC-1_-forward/reverse: 5′-ATCTCTACTTCAACAGCTTTCG/CCTAGCTGCTTTAGCAATCAC-3′) with denaturation at 94°C for 120 s; 35 cycles of denaturation at 94°C for 15 s, annealing at 53°C for 30 s, and elongation for 210 s at 72°C; and a final elongation at 72°C for 1 min. This testing detected *bla*_VCC-1_ in 3 (1.6%) *ctx*-negative, non–O1/O139 *V. cholerae* isolates obtained from waters of the port of Husum, Germany, on the North Sea during 2015. Sanger sequencing confirmed the presence of *bla*_VCC-1_. These 3 isolates belong to the new multilocus ST516. This type is divergent from ST336 only for the novel *pyrC* 150 variant, which was recently deposited in the PubMLST database (https://pubmlst.org).

In conclusion, this study showed the presence of 7 VCC-1 carbapenemase-producing *V. cholerae* at different locations on the coastline of Germany. The *bla*_VCC-1_ flanking genetic sequences were identical in the 4 sequenced *V. cholerae* from Germany and appeared to be different from the strain isolated in Canada, which probably originated in India (Technical Appendix Figure). This finding suggests that *bla*_VCC-1_ was acquired by *V. cholerae* from a yet unknown progenitor on at least 2 occasions. Strains from Germany probably belong to the autochthonous microflora and represent an environmental reservoir of carbapenem resistance in coastal waters. *bla*_VCC-1_–encoding *V. cholerae* might be taken up by mussels, shrimps, and fish and then enter the food chain.

Because carbapenems are needed for treatment of severe infections with multidrug-resistant bacteria in humans, the presence of bacteria with acquired, and thereby potentially transferable, carbapenemases in environments near human activities is a major public health concern (*8*). To date, acquired carbapenemases were detected mainly in clinical isolates and only rarely in environmental and foodborne bacteria (*9*,*10*). Exposure of humans to carbapenemase-producing pathogenic bacteria by ingestion of contaminated food products or by direct contact with contaminated water might pose a threat to public health. Our findings indicate that surveillance for antimicrobial drug resistance should be extended to locations of human activities and foods of aquatic origin.

Technical Appendix Additional information on carbapenemase VCC-1–producing *Vibrio cholerae* in coastal waters of Germany.
