# Supplementary material for: Carbapenemase VCC-1–Producing Vibrio cholerae in Coastal Waters of Germany
Source: Emerg Infect Dis. 2017 Oct;23(10):1735–7. doi: 10.3201/eid2310.161625 (PMC5621562; doi:10.3201/eid2310.161625)
Supplement: Technical Appendix — Additional information on carbapenemase VCC-1–producing Vibrio cholerae in coastal waters of Germany. [file 16-1625-Techapp-s1.pdf]

Article DOI: https://doi.org/10.3201/eid2310.161625

Carbapenemase VCC-1–Producing *Vibrio cholerae* in Coastal Waters of Germany

Technical Appendix

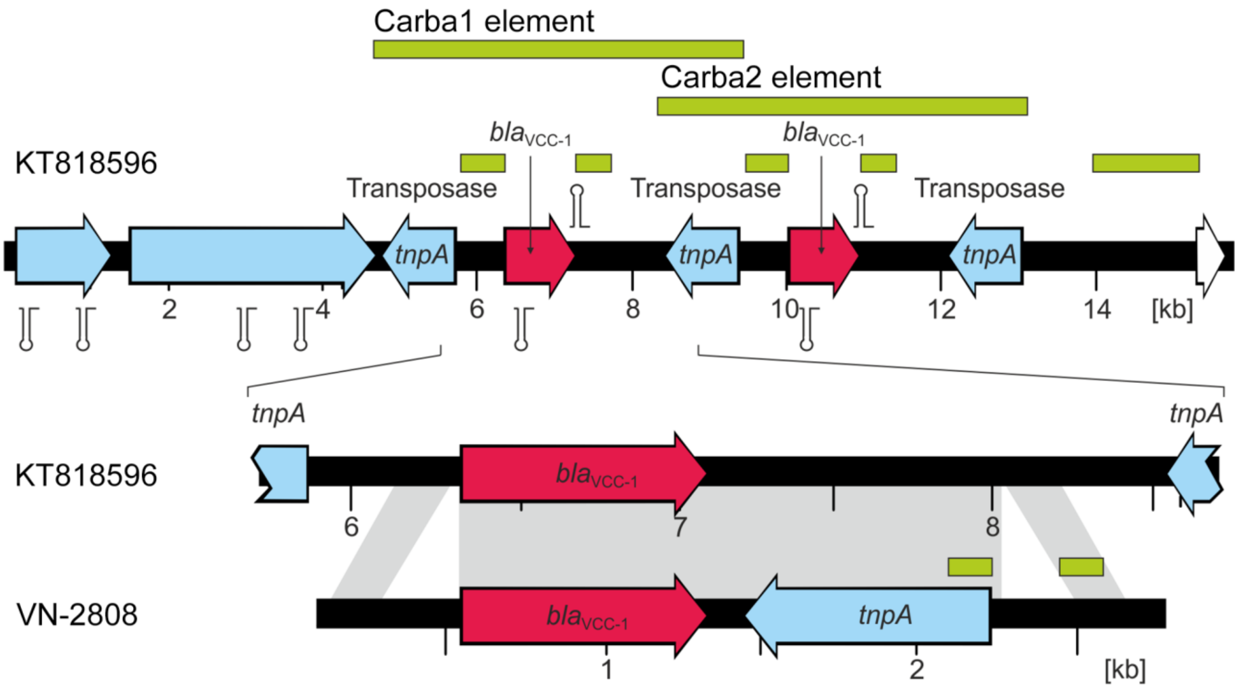

Construct	Insert size, kb	Aztreonam, mm	Meropenem, mm	Imipenem, mm
pVCC-1C	2.7	16	20	17
pVCC-1G	0.9	16	20	16

Technical Appendix Figure. Schematic organization of the *bla*$_{VCC-1}$–encoding region of carbapenemase VCC-1–producing *Vibrio cholerae* VN-2808 in coastal waters of Germany. Identical (100%) DNA sequences of the *bla*$_{VCC-1}$-encoding region of *V. cholerae* N14–02106 (KT818596) and VN-2808 are connected by gray shading. Repetitive sequences are indicated by green bars. Inhibition zone diameters for aztreonam, meropenem and imipenem susceptibility testing conferred by recombinant plasmids pVCC-1C and pVCC-1G in *Escherichia coli* GeneHogs are given. Recombinant plasmids were generated by insertion of PCR products in the multiple cloning site of the vector pIV2. For the *V. cholerae* wild-type strain VN-2808, inhibition zone diameters were 16 mm for aztreonam, 19 mm for meropenem, and 16 mm for imipenem. Carba, carbapenemase.
